# O09 Recurring brachial plexopathy- the zebra among the horses

**DOI:** 10.1093/rap/rkab067.008

**Published:** 2021-10-19

**Authors:** Brooke Mara

**Affiliations:** Cambridge University Hospitals Trust, Cambridge, United Kingdom

## Abstract

**Case report - Introduction:**

A case study of a teenage boy presenting with severe upper limb pain and recurring loss of upper limb function with no clear mechanism of injury. His progress in therapy was initially as expected; however, symptoms would recur despite consistency and compliance with treatment from the patient. This led to a referral for further investigations where a diagnosis of a rare inflammatory neurological condition was made. This case study is relevant for paediatric physiotherapists working in non-inflammatory, musculoskeletal and pain services as it highlights a lesser-known pathology that presents in a similar way to a more common condition.

**Case report - Case description:**

M is a 13-year-old boy that presented with a 5-week history of stabbing pains followed by loss of motor function and sensation in his right arm after swinging a remote. A diagnosis of brachial nerve plexopathy had been suggested.

M had been diagnosed with Hypermobile Ehlers-Danlos Syndrome (hEDS) but was otherwise fit and well with no significant birth, developmental or family history.

He experienced similar episodes of loss of motor function throughout the entire right upper limb following an episode of acute pain aged 4 and aged 12. The episodes were presumed to be a brachial plexus injury following a shoulder subluxation; however, there was no real mechanism of injury to suggest this and symptoms self-resolved after several months in both instances. Age 8 he lost function and sensation in the left arm after a minor pulled elbow, he underwent elbow surgery at another centre to help restore the function of the left arm; however, function didn’t return for approximately 1 year.

On examination he had diminished reflexes throughout the right upper limb and reduced sensation along a C3-8 & T1 distribution. He had a correctable thoracic spine kyphosis with significant medial boarder scapula winging on the right. His right shoulder sat lower than the left and he had muscle atrophy at right supraspinatus, infraspinatus, and serratus anterior and deltoid with tight pectoral muscles.

He was compensating using upper trapezius to achieve 90—100 degrees of shoulder flexion and abduction with 2/5 muscle power. His elbow muscle strength was reduced to 4/5 in all movements on the right. He could only actively extend his right wrist to 30 degrees and only had flickers of active radial deviation. He lacked active finger extension in digits 2-5 and had 0/5 muscle activity at the right thumb.

**Case report - Discussion:**

M underwent exercise therapy with a focus on regaining scapula control in lying and isometric rotator cuff strengthening as he had such significant wasting and was unable to control the upper limb in sitting. We also worked on improving his thoracic spine posture and on active assisted finger and wrist exercises to prevent contractures. I initially provided a sling to be worn at school and in busy environments to prevent any subluxations in view of his significant rotator cuff weakness and history of hEDS. The sling also served as a thoracic posture reminder for M.

After just 2—3 weeks of input and initially making gains in strength and function, M had an episode of severe pain in the right shoulder followed by worsening motor and sensory symptoms. The recurrent nature of episodes and the weak mechanism of injury, led me to discuss M with a consultant. The consultant referred M to genetics where it was discovered he had idiopathic neuralgic amyotrophy (INA; also known as Parsonage—Turner Syndrome), a rare inflammatory neurological disorder. M had the classic signs and symptoms of INA but as he had presented to various different clinicians and centres with each episode a correlation wasn’t made until this latest presentation to pain clinic

**Case report - Key learning points:**

The insubstantial mechanism of injury for his current presentation (motor loss from swinging a remote) led me to probe further into past episodes of his upper limb pain.  This information spurred me to research alternative causes of his symptoms and discuss the case with a consultant who made an onward referral. As physiotherapists we are highly likely to receive referrals for patients like M, with little more information than ‘shoulder pain’ or ‘brachial plexus injury’ given, which is why our subjective is such an important part of the overall assessment.

M’s case highlights how important collating an extensive medical history is to proper investigation and eventual diagnosis. M had a long history of upper limb events for which he had seen a variety of clinicians at various centres. Each event had been treated as an individual episode rather than one larger recurring pattern. Drawing that history together gave a more holistic picture which triggered the referral that identified a diagnosis 8 years after his first presentation to healthcare.

M’s case also highlighted the importance of a good patient—therapist relationship. Motivating a patient with this type of condition is challenging; their progress is not linear and they often have to take steps backwards before they can progress again. This is exceptionally difficult for children and their parents, as it is a frustrating and repetitive cycle. They need to trust that you are giving them the correct therapy and as a therapist you need to trust that the patient is compliant with recommendations and exercise.

Finally, the shoulder rehabilitation for M was, clinically speaking, the same as any other brachial plexus type injury. The main key difference was the need to intermittently take the exercises down a level in the incidence of a new episode of pain and motor loss.

